# Bone marrow cells contribute to seven different endothelial cell populations in the heart

**DOI:** 10.1007/s00395-024-01065-x

**Published:** 2024-07-04

**Authors:** Parisa Shabani, Vahagn Ohanyan, Ammar Alghadeer, Daniel Gavazzi, Feng Dong, Liya Yin, Christopher Kolz, Lindsay Shockling, Molly Enrick, Ping Zhang, Xin Shi, William Chilian

**Affiliations:** 1 Department of Integrative Medical Sciences, Northeast Ohio Medical University, Rootstown, OH 44272 USA; 2 Department of Biomedical Dental Sciences, College of Dentistry, Imam Abdulrahman Bin Faisal University, 31441 Dammam, Saudi Arabia; 3 Institute for Stem Cell and Regenerative Medicine, University of Washington, School of Medicine, Seattle, WA 98109 USA; 4 Hiram College Physics and Computer Science Department, Hiram, OH USA

**Keywords:** Coronary collaterals, Coronary circulation, Bone marrow stem cells, Myocardial ischemia, Single-cell RNA sequencing

## Abstract

Understanding the mechanisms underlying vascular regeneration in the heart is crucial for developing novel therapeutic strategies for myocardial ischemia. This study investigates the contribution of bone marrow-derived cells to endothelial cell populations in the heart, and their role in cardiac function and coronary circulation following repetitive ischemia (RI). Chimeric rats were created by transplanting BM cells from GFP female rats into irradiated male recipients. After engraftment chimeras were subjected to RI for 17 days. Vascular growth was assessed from recovery of cardiac function and increases in myocardial blood flow during LAD occlusion. After sorting GFP^+^ BM cells from heart and bone of Control and RI rats, single-cell RNA sequencing was implemented to determine the fate of BM cells. Our in vivo RI model demonstrated an improvement in cardiac function and myocardial blood flow after 17 days of RI with increased capillary density in the rats subjected to RI compared to Controls. Single-cell RNA sequencing of bone marrow cells isolated from rats' hearts identified distinct endothelial cell (EC) subpopulations. These ECs exhibited heterogeneous gene expression profiles and were enriched for markers of capillary, artery, lymphatic, venous, and immune ECs. Furthermore, BM-derived ECs in the RI group showed an angiogenic profile, characterized by upregulated genes associated with blood vessel development and angiogenesis. This study elucidates the heterogeneity of bone marrow-derived endothelial cells in the heart and their response to repetitive ischemia, laying the groundwork for targeting specific subpopulations for therapeutic angiogenesis in myocardial ischemia.

**Supplementary Information:**

The online version contains supplementary material available at 10.1007/s00395-024-01065-x.

## Introduction

Coronary angiogenesis and arteriogenesis are adaptations to myocardial ischemia that restore myocardial perfusion and mitigate ischemic injury [20, 41, 50, 56]. Ischemia could trigger angiogenesis by upregulating VEGF mRNA expression and stabilizing VEGF mRNA in various tissues [50]. Inducing repetitive ischemia using an external inflatable pneumatic occluder on the coronary artery has been shown to promote collateral blood vessel development in hearts across different animal species [14, 38, 56]. Previous studies have highlighted the ability of bone marrow-derived cells to home to various tissues, express endothelial markers, and contribute to both physiological and pathological neovascularization [2, 25, 54]. Initial investigations in animal models of myocardial infarction demonstrated improved blood flow and heart function following the injection of endothelial progenitor cells [27, 29]. Our previous work indicated that granulocyte-colony stimulating factor mobilizes bone marrow cells, stimulating coronary collateral growth [9], and more recently, we reported the homing of bone marrow cells to heart blood vessels Additionally, we observed that bone marrow stem cells appeared to enhance coronary collateral growth in our model of repetitive myocardial ischemia [5], although the fate of these cells within the heart remained unclear.

Studies in mouse models, such as Cdh5-CreERT2 and Pdgfb-iCreERT2 mice, have shed light on the contribution of endothelial cell expansion to heart neovascularization post-myocardial infarction [36]. While these investigations identified Cdh5-positive and Pdgfb-positive cells in heart neovasculogenesis, they did not directly address the presence of bone marrow-derived cells in the heart. Previous studies using Cd34-CreERT2 mice transplanted into wild-type mice revealed a limited number of Cd34^+^/Cd31^+^ cells in the femoral artery [22]. Due to the heterogeneity and complexity of endothelial cells, the use of specific markers for their detection can potentially result in the oversight of certain populations.

Despite considerable research, the therapeutic efficacy of bone marrow-derived stem cells in ischemic heart disease remains a topic of debate [4, 15, 21, 24, 37, 39, 44, 47, 48]. We opine that a possible reason for this debate is that the basic biology of these cells, and the basis of their healing properties, e.g., differentiation, exosomes, paracrine mechanisms, is not sufficiently delineated, and without this knowledge, implementation of BM cells as a therapy is premature.

In this study, we aimed to elucidate the direct role of bone marrow-derived stem cells in adapting the coronary circulation to myocardial ischemia and tissue hypoxia. To achieve this, we conducted a comprehensive single-cell analysis of bone marrow cells from both the bone and the heart to characterize the entire population of bone marrow-derived cells that home to the heart under both homeostatic and ischemic conditions.

## Methods

### Rats

Animal procedures were performed in accordance with the National Institutes of Health Guidelines for the Care and Use of Laboratory Animals and approved by the Institutional Animal Care and Use Committee of Northeast Ohio Medical University. Rats were kept on a 12/12-h dark–light cycle in a temperature-controlled room and provided ad libitum standard chow and water. Sprague–Dawley (SD) rats (2-month-old) were purchased from Charles River Laboratories. Transgenic SD rats which ubiquitously express enhanced GFP under control of the human ubiquitin-C promoter with the woodchuck hepatitis virus posttranscriptional regulatory element (WRE) were purchased from Rat Resource and Research Center, University of Missouri.

### Bone marrow transplantation

For bone marrow transplantation, SD rats were irradiated at lethal dose by a split dose of 500 rad (4 h between doses) from an X-ray irradiator (RS2000, Rad Source Technologies, Suwanee, GA). On the same day, bone marrow (BM) was isolated from donor rats after euthanasia using intraperitoneal (IP) injection of Fatal Plus (0.2–0.3 ml/kg) (Covetrus, OH). BM cells were freshly isolated from femurs and tibias of rats by removing the caps of the bones, flushing and resuspending the bone marrow in FBS/PBS (50%). The BM cells were centrifuged at 350×*g* for 5 min. Mononuclear BM cells were obtained after using red blood cell (RBC) lysis buffer (eBioscience) to remove the RBCs, filtered through a 40-μm cell strainer (Fisher Scientific, Pittsburgh, PA), and washed with PBS by centrifugation at 350×*g* for 30 min. Anesthesia was induced in the recipient rats with a mixture of 4% isoflurane (Covetrus) and oxygen in a sealed chamber. After induction, anesthesia was maintained with 3% isoflurane during the bone marrow transplantation. Bone marrow cells (5 × 10^7^ cells) in phosphate-buffered saline (PBS) were transplanted through a lateral femoral incision of irradiated rats. We conducted two sets of experiments. In the first set, we transplanted whole GFP bone marrow cells from GFP transgenic donor rats into the femur of irradiated wild-type recipients. In the second set, we performed a Cd34 depletion experiment. We isolated and sorted BM cells for Cd34 from donor rats, and subsequently transplanted the Cd34-negative population into irradiated wild-type rats. Rats were given acid antibiotic water (supplemented with 150 mg/L neomycin, 5 mg/L bacitracin and pH = 3) and irradiated food for four weeks.

### Echocardiographic analysis of cardiac function

A Siemens ultrasound imaging system (Sequoia Acuson C512; Siemens Medical Systems USA Inc., Mountain View, CA) was used for two-dimensional echocardiography and contrast echocardiography. Contrast imaging was performed through infusion of a contrast agent (lipid-shelled microbubbles) into the tail vein through a PE-50 catheter. The animals were anesthetized using 4% isoflurane. Anesthesia was maintained with 2% isoflurane during echocardiography. Measurements of cardiac function and myocardial blood flow (MBF) including collateral flow (collf) and normal zone flow were performed under basal conditions (Baseline) and after inflating the occluder (Inflated). Rats were scanned in a supine position and the animals were kept warm on a heated platform. Systolic left ventricular function was assessed using a high-frequency linear-array transducer (15L8). Standard two-dimensional M-mode echocardiographic measurements were performed in parasternal long axis views. For the evaluation of left ventricular systolic function, we measured ejection fraction (EF%) and Fractional Shortening (FS%) of the apex.

To quantify myocardial blood flow, we employed a contrast imaging technique. This involved the preparation of a contrast agent through sonication of an aqueous suspension saturated with decafluorobutane gas. The suspension contained distearoylphosphatidylcholine (2 mg/mL) and polyoxyethylene-40-stearate (1 mg/mL). Long axis images of the left ventricle were obtained for perfusion imaging. Images were stored after a high-energy pulse to destroy the microbubbles and for several seconds after destruction to establish refilling of the chamber and ventricular wall. To do data analysis on contrast echo images, we defined regions of interest (ROI) within the anterolateral wall and cavity in the long axis view of LV; they were positioned in the apex as well as the base of LV. A curve of signal intensity over time was obtained in the ROI and relative blood volume (RBV) was calculated as the ratio of myocardial to cavity signal intensity. Myocardial blood flow was calculated by multiplying RBV by the initial slope of the curve, which corresponds to the blood volume exchange frequency.

### Repetitive ischemia (RI) model

To induce RI, we implanted a pneumatic occluder over the left anterior descending coronary artery (LAD) as described previously [56]. In brief, after doing left thoracotomy at the 3–4 intercostal space, pericardium was incised. A 6–0 suture was threaded around the proximal portion of the LAD and a balloon occluder was tied onto the surface of the heart. The correct position of the occluder was checked by blanching of the LV apex and ST elevation in ECG upon occluder inflation. The tubing portion of the occluder was tunneled through the chest wall and the chest was closed. The chest cavity was then evacuated of air. The tubing was tunneled subcutaneously and externalized between the scapulae and secured with a Teflon button. The occluder was inflated by a computer-controlled system for 40 s every 20 min, for 2 h and 40 min, followed by 5 h and 20 min of rest. This 8-h cycle was repeated 3 times a day over17 days. Anesthesia was induced by a mixture of 4% inhaled isoflurane and oxygen. After induction and intubation, rats were ventilated with a mixture of 3% isoflurane and oxygen by a ventilator (HALLOWELL EMC) during surgery.

### Single-cell RNA sequencing

Single-cell RNA sequencing (RNA seq) was performed on GFP^+^ PI^−^ cells isolated from the hearts and bones of Control rats (*n* = 2) and RI rats (*n* = 2). The Control and RI rats were male wild-type rats that received whole GFP^+^ BM cells from female GFP rats. However, the RI rats underwent repetitive ischemia. To harvest the hearts and bone marrow, rats were deeply anesthetized using 4% isoflurane and euthanasia was accomplished after removal of the heart (exsanguination). We used 10X genomics platform for doing single-cell RNA sequencing. Bone marrow cells were isolated from the rat bones by flushing bones as described above. Bone marrow cells were isolated from the rat hearts by heart digestion. We used a Langendorff-free protocol to digest the heart and the buffers components as described previously [1]. For heart digestion, the rat’s chest was opened to expose the heart, and the descending aorta and inferior vena cava were both cut. EDTA buffer (20 ml) was injected into the right ventricle to clear the heart of blood cells. The ascending aorta was then clamped. The clamped heart was transferred to a dish containing EDTA. After injecting 40 ml of EDTA buffer through the LV wall, the heart was transferred to the dish containing perfusion buffer. Perfusion buffer (20 ml) was then injected into the LV via the same point. Then the heart was transferred to the dish containing collagenase buffer (collagenase 2 (Worthington, LS004176, 0.5 mg/ml), collagenase 4 (Worthington, LS004188, 0.5 mg/ml) and protease XIV (Sigma, P5147, 0.05 mg/ml)), and the LV was injected through the same point with 80 ml collagenase buffer. The enzymatic digestion was done on a heating pad for 30–45 min. The heart is then transferred to the new dish of collagenase buffer. After removing atria and any leftover fatty tissues as well as pericardium, the tissue is then teased apart into small pieces. The heart pieces were gently triturated using a 5 ml pipette and transferred into 15 mL of collagenase-containing medium and digested in the 37 °C incubator for 15 min with occasional mixing using a 5 mL pipette. Stop solution was then added to the suspension to inhibit further enzymatic digestion. The tissue-cell suspension was passed through a 100 μm strainer and was centrifuged at 25×*g* for 1 min (without brakes) to pellet the cardiomyocytes. The supernatant was removed and centrifuged at 350×*g* for 5 min. After adding DMEM medium to the pellet, the cell suspension was passed through a 40 μm strainer and prepared for sorting. Sorting of live GFP expressing cells in cell suspensions isolated from the heart and bone was conducted on a FACSAria Fusion flow cytometer with FACSDiva software (Becton Dickinson, San Jose, CA). Nonviable cells in cell suspension samples were stained with propidium iodide (PI) for exclusion from sorting. Gated PI^−^ GFP^+^ cells were sorted out for single-cell RNA sequencing.

#### Chromium 10X library preparation

After cell sorting, single cells were manually counted by Trypan blue exclusion using hemocytometer. Cell suspension was diluted to 1600 cells per microliter resulting in recovery of 1000 cells per microliter and loaded on the Chromium Controller (10X Genomics) with a targeted cell 3000–4000 cells per sample. 3′ gene expression libraries were constructed according to the manufacturer’s instructions using Chromium Single Cell 3′ v3 Reagent Kit (10X Genomics). Quality control of cDNA libraries were performed using Bioanalyzer High Sensitivity DNA Analysis (Agilent)). Libraries were sequenced using NextSeq 550 Sequencing System.

#### Demultiplexing and transcriptome mapping

Sequencing outputs were demultiplexed to convert base call files (BCL) files to FASTQ format. The cellranger count pipeline was used to align sequencing reads to the mRatBN7.2 reference genome and generating the count matrix for further analysis.

#### Filtering and clustering of scRNA-seq data

Seurat package (version 4.0.6) was used for quality control and data analysis of the single-cell expression matrix [16]. Cells with unique feature counts over 2,500 or less than 200 were excluded. We also filtered out the cells that had greater than 15–30% mitochondrial gene ratio as well as the cells that had expression of any of Y chromosome genes. Quality control metrics for BM and BMH cells are shown in Supplementary Fig. 2a and b. We next performed log normalization of the counts with the ‘‘NormalizeData’’ function. We next calculated highly variable features using the ‘‘FindVariableGenes’’ function and used them for PCA analysis. We used ‘FindClusters’’ function which applies a graph-based clustering approach to cluster the cells. Clusters were visualized using a 2-dimensional t-distributed stochastic neighbor embedding (t-SNE) algorithm with the ‘‘RunTSNE’’ function. Clusters were annotated using the abundance of canonical marker genes.

#### Differential expression genes analysis

Differential expression analysis was performed using the ‘‘FindMarkers’’ function of the Seurat package. As a default, Seurat performs differential expression using the non-parametric Wilcoxon rank sum test. We identified DEGs among different cell clusters. We also found DEGs between BMH-Control and BMH-RI endothelial cells. Adjusted P value, corrected for Bonferoni, was used for comparing multiple groups and P value was used for comparing two groups. *P* values of < 0.05 was considered to be significant. We used “aggregate_gene_expression” function in Monocle 3 to visualize the average of gene expression per group or endothelial cell subclusters. This function makes a matrix with aggregated expression values for genes of each group. The values are divided by cell size factors before aggregation. Aggregated expression values were scaled and represented by Z score.

#### Gene ontology (GO) analysis

To perform the Gene Ontology analysis on the endothelial cells of Control and RI groups, the top 100 significant DEGs with a log-transformed fold change > 0 (upregulated gernes) were searched in Metascape. (3). The top six significantly enriched biological processes for each group were selected and were visualized with the ggplot2 R package.

#### Finding modules of co-expressed genes

We used Monocle 3 to find modules of co-regulated genes [7, 8, 31, 40, 57, 58, 61]. We first generated a monocle cell_data_set. Monocle 3 uses Louvain community analysis to group the genes into modules. We show aggregate expression of all genes of each module across all endothelial cell subclusters and in BMH-Control and BMH-RI groups as well. To visualize cell-to-cell variations, the expression of modules was applied in the tSNE plot.

#### Cell–cell communication analysis

Cell–cell communication was assessed using CellChat package [23]. After creating CellChat object using single-cell RNA seq gene expression matrix, CellChatDB database was used to identify the cell–cell communications. Over-expressed ligand–receptor interactions of cells were determined. Each interaction is assigned with a probability value. CellChat uses ‘trimean method for calculating the average gene expression per cell group. We used 10% truncated mean to calculate the average gene expression. Number of interactions or interaction strength among different cell populations were compared in BMH-RI and BMH-Control groups. Upregulated and downregulated signaling ligand–receptor pairs in the BMH-RI compared to BMH-Control was determined based on the differential gene expression analysis.

#### Immunohistochemistry and confocal microscopy

Rat hearts were perfused with PBS via aorta until heart was cleared of the blood. Then Paraformaldehyde (PFA) 4% was perfused, and the heart was removed and kept in 4% PFA for 4 h in the fridge. The samples were rinsed three times with PBS on a shaker with constant shaking. Next the hearts were immersed in 15% sucrose overnight followed by overnight immersion in 30% sucrose. The tissues were then embedded in OCT and frozen on dried ice. The tissues were stored in − 80 till cryosectioning. Cryosections were produced at a cryostat (LEICA CM 1950). To do staining, the sections were permeabilized and blocked for an hour in freshly prepared blocking/permeabilization solution (0.3% Triton X-100 and 10% goat serum in PBS) at room temperature. Then, they were incubated with primary antibody overnight at 4 °C. The next day, the primary antibodies were washed in PBS. Secondary antibodies were incubated for an hour at room temperature. After washing the secondary antibody and staining with 4′,6-diamidino-2-phenylindole (DAPI) (Sigma, MBD0015-1M), imaging was performed using a confocal microscope. All quantitative evaluations were performed with Fluview and ImageJ software. The following primary and secondary antibodies with indicated dilutions were used: Reca-1 (Abcam, ab264524, 1:100), Lyve1 (Novus Biological, NB600-1008, 1:100), Isolectin GS-IB4 From Griffonia simplicifolia, Alexa Fluor™ 594 Conjugate (Thermo Fisher Scientific, I21413, 1:200), Goat anti-Mouse secondary Antibody, Alexa Fluor™ 647 (Thermo Fisher Scientific, A-21235, 1:500).

#### Statistical analysis

Data are presented as mean $\pm$ standard deviation (SD). Statistical analyses were performed in PRISM software (GraphPad 9). Student’s *t* test was used to compare the difference between two groups. One-way analysis of variance (ANOVA) with Bonferroni corrections post hoc was used to compare the difference among groups. Significant differences were defined by *P* < 0.05 (*) or (#), *P* < 0.01 (**) or (##), *P* < 0.001 (***) or (###), *P* < 0.0001 (****) or (####).

## Results

### Cardiac function and myocardial blood flow after repetitive ischemia

**Fig. 1:**
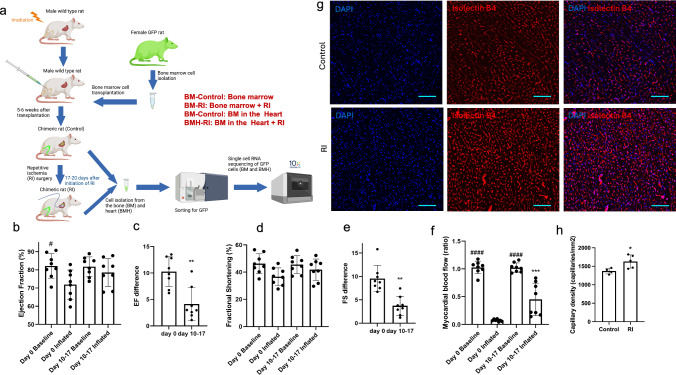
A schematic of the experimental protocol and echocardiographic measurements of cardiac function. **a** Bone marrow was harvested from the femur of female donor transgenic rats expressing green fluorescent protein (GFP). Recipient irradiated wild-type male rats received GFP^+^ bone marrow cells, injected into the femur. After 5–6 weeks, an occluder was implanted on LAD and the rats were subjected to repetitive ischemia (RI) for 17 days to stimulate blood vessel growth in the heart. Measurements of heart function and myocardial blood flow were made at day 0 and at the end of the RI protocol to evaluate the extent of coronary collateral growth. To do single-cell RNA sequencing, after killing, the heart was enzymatically treated to disassociate cells. GFP^+^ cells from the bone and heart of Control and surgeried rats were used for single-cell study. **b** Ejection fraction (EF%) between baseline and inflated conditions on days 0 and 17 post initiation of inflation (*n* = 8). **c** Difference of EF between baseline and inflated conditions on days 0 and 17 post initiation of inflation (*n* = 8). **d** Fractional shortening (FS%) between baseline and inflated conditions on days 0 and 17 post initiation of inflation (*n* = 8). **e** Difference of FS between baseline and inflated conditions on days 0 and 17 post initiation of inflation (*n* = 8). **f** Ratio of myocardial blood flow in ischemic zone to normal zone under baseline and inflated conditions on days 0 and 17 post initiation of inflation (*n* = 8). **g** Representative image of rat heart sections stained for isolectin B4 counterstained with DAPI. Scale bars represent 100 μm. **h** Quantification of capillaries in the BMH-Control (*n* = 4) and BMH-RI (*n* = 5) groups. A paired Student’s *t* test was used to compare the difference of EF and FS between day 0 and 17 after inflation and also the capillary density in the BMH-Control and BMH-RI groups. One-way analysis of variance (ANOVA) was used to compare myocardial blood flow among baseline and inflated conditions on day 0 and 17 after inflation. *indicates difference of myocardial blood flow of inflated condition between day 0 and day 17. ^#^indicates difference of myocardial blood flow between baseline and inflated conditions

Figure 1a illustrates the schematic of our experimental protocol. In our in vivo repetitive ischemia (RI) model, occluder inflation led to a decrease in LV ejection fraction (EF) and fractional shortening (FS) compared to baseline. Notably, the EF difference between baseline and inflated conditions significantly decreased by day 17 compared to day 0, indicating an improvement in heart function within the ischemic area (Fig. 1b, c). This improvement was consistent with the FS measurements (Fig. 1d, e). To gain insight into the degree of collateral growth following RI, we measured myocardial blood flow (MBF) on day 0 and 17 in the base (normal zone) and apex (ischemic zone) of the left ventricle at baseline and during occlusion (inflation). Contrast images show microbubbles in the left ventricular cavity and anterior wall, indicating perfusion, as denoted by the bright signals from the echogenic material (Supplementary Fig. 1a). On day zero, during inflation, the apex received little flow as evidenced by the dark area in the anterior wall (ischemic zone). After 17 days of RI, increased vascular growth was observed, as indicated by the greater number of echogenic microbubbles in the ischemic zone (Supplementary Fig. 1a). MBF analysis, indicated by the ratio of MBF in the ischemic zone to the normal zone, exhibited a significant decrease under inflated conditions compared to baseline on day 0. However, by day 17 after repetitive ischemia (RI), the decrease in MBF ratio was markedly attenuated in the inflated condition, with the MBF ratio increasing approximately fourfold (Fig. 1f).

Furthermore, we assessed capillary density in rat heart sections from two groups: control rats that underwent bone marrow transplantation (Control) and rats subjected to RI for 17 days after bone marrow transplantation (RI). Our results demonstrated a significantly higher capillary density in the RI group compared to the Control group (Fig. 1g, h).

### Single-cell RNA sequencing of whole bone marrow cells from the bone (BM) and BM fraction of rat hearts (BMH)

**Fig. 2:**
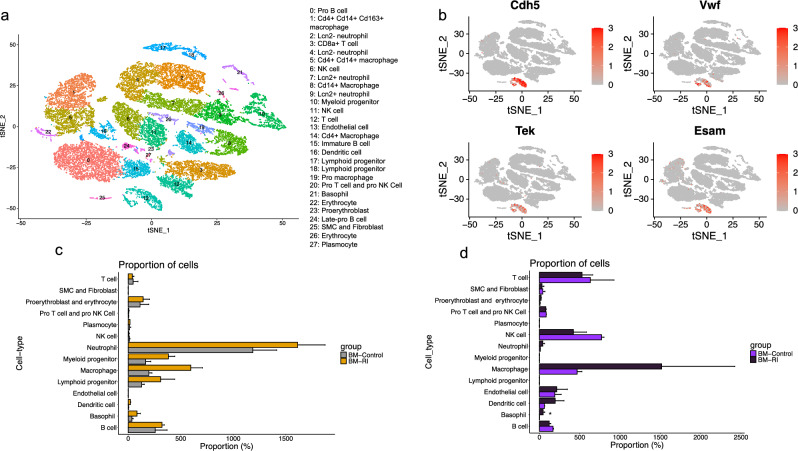
Proportion of different cell types in different groups. **a** t-Distributed stochastic neighbor embedding (t-SNE) plot of the pools single-cell RNA sequencing data of whole BM cells isolated from the bone and heart. **b** t-SNE representation of signature genes for endothelial cells (Cdh5, Vwf, Tek, and Esam) in whole population. **c** Bar graph showing relative fraction of different cell types of BM. **d** Bar graph showing relative fraction of different cell types of BMH. An unpaired Student’s *t* test was used to compare the difference of cell proportion between Control and RI groups

We conducted single-cell RNA sequencing on GFP^+^/PI^−^ BM cells isolated from both bones (BM) and hearts (BMH) of BM transplanted rats (Supplementary Fig. 1b) at day 17 post initiation of RI. The 10 × Genomics Chromium platform was employed to elucidate gene expression and phenotype of GFP + cells. A total of 31,512 cells were sequenced, from which 24,103 qualified cells were retained after quality control filtering for the number of detected unique genes and mitochondrial read counts using Seurat V4 (Supplementary Fig. 2a and 2b). Subsequently, a graph-based clustering approach was employed to identify cell clusters, and the populations of cells belonging to these clusters were visualized in a t-distributed stochastic neighbor embedding (t-SNE) dimensionality reduction plot. Differential gene expression (DEG) analysis was performed to determine the cell types represented by each cluster, and the clusters were annotated based on the expression levels of canonical cell-type-specific markers. This analysis revealed 28 cell clusters including 3 clusters of B cells, 3 clusters of T cells, 2 clusters of NK cells, one cluster of dendritic cells, one cluster of basophil cells, 5 macrophage clusters, 4 clusters of neutrophil cells, one cluster of plasmacytes, one cluster of myeloid progenitor cells, 2 cluster of lymphoid progenitor cells, one cluster of endothelial cells, 3 clusters of erythroblasts and erythrocytes, one cluster of smooth muscle cell and fibroblast (Fig. 2a and Supplementary Fig. 2c). Comparison of the abundance of different cell populations between the two groups indicated that although not statistically significant, macrophages, neutrophils and basophils showed an increase in the BMH-RI compared to BMH-Control. Conversely, B cells, T cells, and NK cells exhibited a decreasing trend in BMH-RI compared to BMH-Control (Fig. 2d). Consistent with a trend of higher abundance of myeloid derived cells (macrophages, neutrophils and basophils) in the BMH-RI, a higher proportion of myeloid progenitors was observed in BM-RI compared to BM-Control (Fig. 2c). Furthermore, the proportion of proliferating macrophages in BMH-RI was increased compared to the BMH-Control (Supplementary Fig. 3b).

### Characterization of BM-derived endothelial cells in the heart

**Fig. 3:**
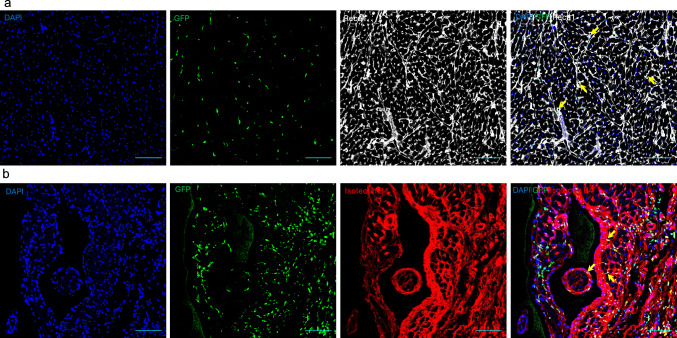
Presence of bone marrow (BM)-derived endothelial cells in the left ventricle. Representative image of rat heart sections stained for rat endothelial cell antigen-1 (Reca-1) (**a**) and isolectin B4 (**b**) counterstained with DAPI. The BM-derived cells are GFP positive. Scale bars represent 100 μm

**Fig. 4:**
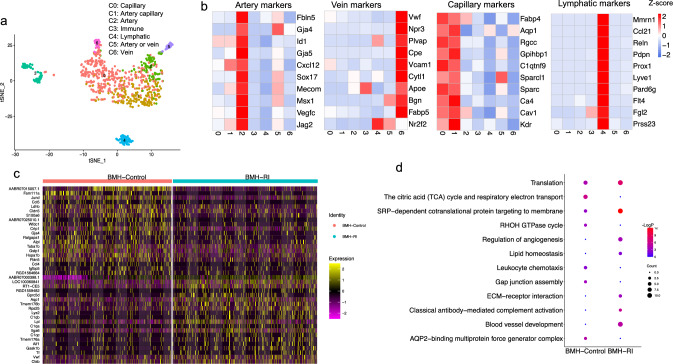
Heterogeneity of BM-derived endothelial cells from the heart of Control and RI rats. **a** Distributed stochastic neighbor embedding (t-SNE) plot visualization of endothelial cells showing different endothelial cell subtypes. **b** Gene expression heatmap of canonical markers for endothelial cell subtypes. **c** Expression of top differentially expressed genes across the endothelial cells in each group. **d** Representative GO term enrichment of genes significantly upregulated in the endothelial cells from BMH-Control and BMH-RI. Color scale: red, high expression; blue, low expression

Notably, we identified a cluster (cluster 13) exhibiting high expression of endothelial markers which almost exclusively belongs to BM cells in the heart (BMH) (Fig. 2b and Supplementary Fig. 4). These results were confirmed by histology showing GFP^+^ cells expressing endothelial markers such as rat endothelial cell antigen-1 (RECA-1) and Isolectin B4 (Fig. 3 and Supplementary Fig. 5). To explore the heterogeneity of BM-derived endothelial cells (ECs), we employed a graph-based subclustering approach on the entire BMH EC population, identifying a total of 7 distinct EC subpopulations (Fig. 4a) using top 100 marker genes for each subcluster. Subclusters were annotated based on the expression of canonical markers for different EC subtypes (Fig. 4a). In subclusters 0 and 1, previously reported capillary markers for heart including Rgcc and Ca4 were enriched (Fig. 4b). Artery markers including Gja4 and Gja5 were predominantly expressed in the subcluster 2 (Fig. 4b). We observed expression of capillary and some artery markers in the subcluster 1 (Fig. 4b). We designated subcluster 0, 1 and 2 as capillary, artery, and capillary artery, respectively. Additionally, we identified a subcluster (subcluster 3) characterized by the expression of Ptprc (Cd45) (Supplementary Fig. 6a). This cluster exhibited the lowest expression of endothelial markers (Supplementary Fig. 6b) and was designated as immune ECs. Subcluster 4 showed high expression of lymphatic EC markers including Prox1 and Pdpn and was classified as lymphatic ECs (Fig. 4b). Subcluster 6 expressed vein markers such as Bgn and was classified as venous ECs (Fig. 4b). Subcluster 5 exhibited expression of both artery and vein markers and was annotated as artery or vein ECs (Fig. 4b).

### Angiogenic profile of BM-derived endothelial cells after RI

Heatmap analysis of the top 20 markers using cells and features PCA scores revealed distinct signatures of ECs between BMH-Control and BMH-RI groups (Fig. 4c). Subsequently, we explored the biological implications of repetitive ischemia (RI) using Gene Ontology (GO) and pathway analyses (http://metascape.org/) for two groups. The upregulated genes in BMH-RI compared to BMH-Control exhibited greater enrichment in processes related to blood vessel development, regulation of angiogenesis, lipid homeostasis, and ECM receptor interaction, indicating a more angiogenic profile of ECs in BMH-RI group (Fig. 4d). The expression levels of upregulated angiogenic markers in BMH-RI compared to BMH-Control are depicted in Supplementary Fig. 6c. Conversely, the upregulated genes in BMH-Control compared to BMH-RI, showed greater enrichment in processes related to the TCA cycle and respiratory electron transport, RHOH GTPase cycle, Gap junction assembly and leukocyte chemotaxis (Fig. 4d).

### Characterization of BM-derived endothelial cells heterogeneity

**Fig. 5:**
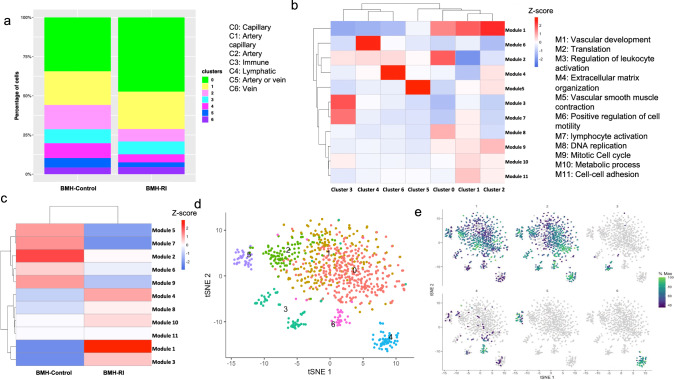
Co-expression modules in BM-derived endothelial populations. **a** Stacked bar graph showing relative fraction of endothelial cells subtypes in BMH-Control and BMH-RI groups. **b** Heatmap and hierarchical clustering of co-expression modules across different endothelial cells subtypes. **c** Heatmap and hierarchical clustering of co-expression modules in BMH-Control and BMH-RI groups. **d** Distributed stochastic neighbor embedding (t-SNE) plot visualization of endothelial cells showing different endothelial cell subtypes. **e** t-SNE plot showing expression of the modules which are specific to certain endothelial cell subtypes

**Fig. 6:**
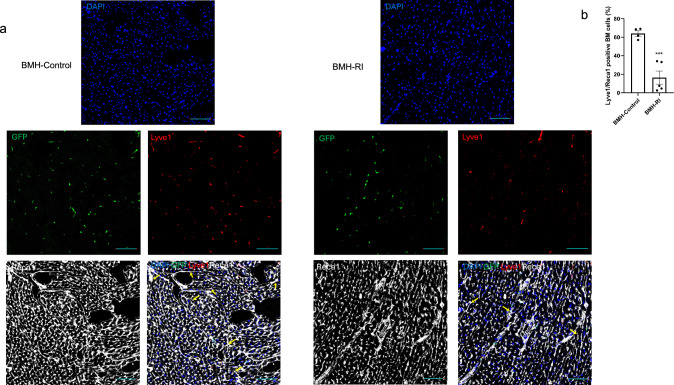
Proportion of BM-derived lymphatic endothelial cells. **a** Representative image of rat heart sections stained for rat endothelial cell antigen-1 (Reca-1) (white), lymphatic vessel endothelial hyaluronan receptor 1 (Lyve1) (red), green fluorescent protein (GFP) (green) counterstained with 4',6-diamidino-2-phenylindole (DAPI) in BMH-Control and BMH-RI groups. Arrows indicate GFP^+^/Reca-1^+^/Lyve1^+^ cells. **b** Quantification of GFP^+^/Reca-1^+^/Lyve1^+^ cells in the BMH-Control (*n* = 4) and BMH-RI (*n* = 5) groups. An unpaired Student’s *t* test was used to compare the difference of cell proportion between Control and RI groups. Scale bars represent 100 μm

We assessed the proportion of EC types in BMH-Control and BMH-RI groups. Although not statistically significant, the BMH-RI group exhibited an increasing trend in the abundance of capillary ECs (subcluster 0). In contrast, the BMH-Control group showed an increasing trend in the proportion of artery and lymphatic endothelial cells (Fig. 5a). When comparing the percentage of lymphatic ECs between the Control and RI groups in heart sections, it revealed a higher percentage of lymphatic ECs in the Control group (Fig. 6).

To identify co-expression modules in the total BM-derived endothelial cell population, we utilized Monocle 3 and discovered 11 modules (Fig. 5b). While modules 8, 9, 10, and 11 are shared across all EC subclusters, some modules are specific to certain subclusters. Gene Ontology (GO) analysis revealed that each module was enriched for genes involved in specific biological processes. We named each module based on its most enriched biological process. Module 1, mainly associated with the vascular development pathway in GO enrichment analysis, was upregulated in capillary, arterial, and capillary arterial subclusters (Fig. 5b). Comparing the expression of modules between the BMH-Control and BMH-RI groups showed a high enrichment of module 1 in the BMH-RI group compared to the BMH-Control group (Fig. 5c). Leukocyte activation module was enriched in the immune ECs (subcluster 3) (Fig. 5b, d, e). Venous ECs exhibited enrichment of module 4 related to extracellular matrix organization (Fig. 5b, d, e). Subcluster 5 demonstrated high expression of module 5 related to the vascular smooth muscle contraction pathway (Fig. 5b, d, e).

### Heterogeneity of EC subpopulations in metabolic gene signatures

**Fig. 7:**
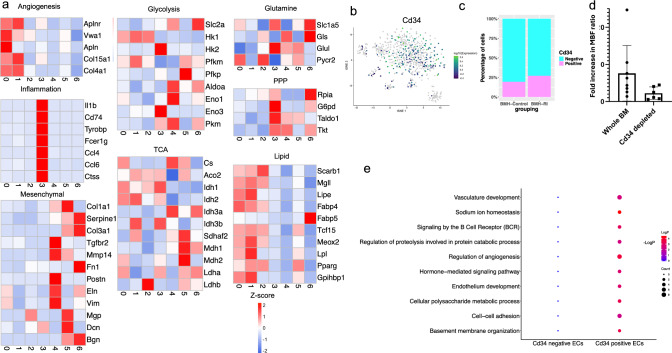
Expression of different metabolic signatures in BM-derived endothelial populations. Heatmaps showing expression of representative genes involved in angiogenesis, inflammation, mesenchymal activation, glycolysis, tricarboxylic acid (TCA), glutamine, pentose phosphate pathway, lipid metabolism (**a**). **b** Distributed stochastic neighbor embedding (t-SNE) plot visualization of endothelial cells expressing Cd34. **c** Stacked bar graph showing relative fraction of Cd34^+^ and Cd34^−^ endothelial cells in BMH-Control and BMH-RI groups. **d** The fold increase in myocardial blood flow ratio in ischemic zone to normal zone under inflated condition on day 17 post initiation of inflation in the rats which received whole BM cells compared to those which received Cd34-depleted BM cells. **e** Representative GO term enrichment of genes significantly upregulated in the Cd34^+^ and Cd34^−^ endothelial cells

We further investigated the potential heterogeneity in metabolic gene expressions across different EC subclusters. Differential gene expression analysis showed that, although there were no discernible differences in the tricarboxylic acid (TCA) cycle, glycolytic, and glutamine pathways among different subclusters (Fig. 7a), capillary and arterial capillary ECs exhibited upregulation in the expression of genes involved in lipid metabolism, including Gpihbp1, Lpl, Tcf15, and Fabp4 (Fig. 7a). Moreover, some of the pentose phosphate pathway (PPP) genes, such as G6pd and Taldo1, were enriched in immune ECs (Fig. 7a).

As previously discussed, gene ontology analysis revealed an enrichment of TCA and respiratory electron transport in the BMH-Control ECs and a lipid homeostasis pathway in the BMH-RI ECs (Fig. 4d). Violin plots illustrate the expression of upregulated genes involved in the TCA cycle (Supplementary Fig. 7a) and lipid homeostasis (Supplementary Fig. 7b) in BMH-Control and BMH-RI ECs, respectively.

Subsequently, we examined the expression patterns of angiogenesis, inflammatory, and mesenchymal markers across various EC subclusters. We detected elevated expression levels of angiogenesis markers within the capillary and artery capillary subclusters, as depicted in the heatmap (Fig. 7a).

Inflammatory genes exhibited enrichment primarily in the immune ECs, coinciding with an enrichment of the leukocyte activation module. Additionally, venous ECs and subcluster 5 displayed enrichment of specific mesenchymal markers, such as Col3a1, Fn1, and Serpine 1 (Fig. 7a). The heightened expression of mesenchymal markers (Fig. 7a), alongside reduced expression of endothelial markers primarily in subcluster 5 (Supplementary Fig. 6b), hints at a potential trend toward endothelial to mesenchymal transition within this subcluster.

### ***Role of CD34***^+^***endothelial cells in angiogenesis and collateral blood flow regulation***

We analyzed the expression of Cd34 in our BM-derived EC population and found that 23.5% of all BM-derived ECs express Cd34 (Fig. 7b, c). We evaluated the DEGs in both Cd34^+^ and Cd34^−^ ECs. A heatmap illustrates the top 20 DEGs in the Cd34^+^ and Cd34^−^ BM-derived ECs (Supplementary Fig. 7c). Gene ontology analysis of upregulated DEGs revealed enrichment in vascular development and regulation of angiogenesis in Cd34-positive ECs (Fig. 7e).

To elucidate the role of Cd34^+^ cells in angiogenesis and collateral growth, we conducted another set of experiments wherein we transplanted whole BM cells or Cd34-depleted BM cells to irradiated wild-type mice. After inducing RI for 17 days, we assessed myocardial blood flow ratio in both groups. Our finding showed a significantly lower fold increase in MBF ratio in the rats that received the CD34-depleted BM cells compared to the rats that received the whole BM cells (Fig. 7d).

### Alteration of cell–cell communication pattern after RI

**Fig. 8:**
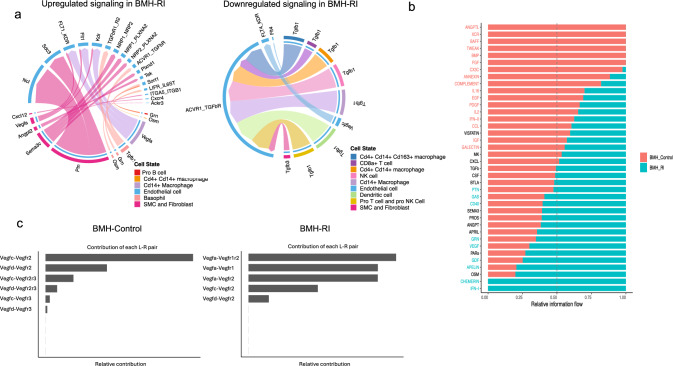
Cell–cell communication among BM-derived cell populations in the heart. **a** Chord diagram showing the upregulated and downregulated ligand–receptor interactions in the BMH-RI endothelial cells compared to the BMH-Control endothelial cells. Cell–cell communication is indicated by the edges. Edge colors are consistent with the colors of sender cells and edge weights indicate the interaction strength. **b** Significant signaling pathways ranking based on the differences in the overall interaction strength between BMH-Control and BMH-RI groups. The top signaling pathways enriched in BMH-Control are red and the ones enriched in BMH-RI are blue. **c** Bar graph showing the contribution of each ligand–receptor pair to the overall signaling pathway in BMH-Control and BMH-RI groups

We then performed differential expression analysis between BMH-Control and BMH-RI for each cell cluster to obtain the upregulated and downregulated signaling based on the expression of ligands in the sender cells and receptors in the receiver cells using CellChat. The up- and downregulated ligand–receptor pairs between endothelial cells as a target and other BM-derived cell populations as sources were visualized using Chord diagram. Downregulated signaling pathways are Tgfb1–ACVR1–TGFbR and Vegfc–KDR–FLT4 in the BMH-RI endothelial cell population (Fig. 8a). One of the prominent up-regulated signaling pathways is Ptn/Sdc3/Ncl from SMC and fibroblasts. Of note, BMH-RI endothelial cells showed enrichment of Vegfa/Kdr/Flt1 signaling (Fig. 8a). Macrophage as well as SMC and fibroblasts showed upregulation of Vegfa expression consistent with angiogenic profile in the BMH-RI endothelial cells (Fig. 8a). Figure 8b shows enrichment of Vegf signaling in BMH-RI compared to the BMH-Control. Figure 8c shows contribution of each ligand–receptor pair to the overall VEGF signaling pathway in BMH-Control and BMH-RI groups. The BMH-Control shows high enrichment of Vegfc signaling, but it does not show contribution of Vegfa. In addition to endothelial, and SMC and fibroblast clusters, the macrophage population also express Vegf to influence BMH-RI cells. The BMH-Control ECs exhibit autocrine secretion of Vegfc and paracrine production of Vegfd by SMC and fibroblast. The BMH-RI ECs exhibit autocrine secretion of Vegfc, receive Vegfc and Vegfd from SMC and fibroblast and also Vegfa from macrophage and to a lesser extent from SMC and fibroblast cells (Supplementary Fig. 8).

### BM-derived SMC and fibroblast population

Since SMC and fibroblast cluster is the closest cluster to endothelial cells and appeared to have strongest communication to endothelial cells, we examined the SMC and fibroblast clusters. We identified 3 subclusters and did DEG analysis and used marker genes from the literature to annotate them (Supplementary Fig. 9a). Subcluster 1 highly expressed fibroblast markers and was assigned as fibroblast. SMC markers are highly enriched in the subcluster 2 (SMC subcluster). We could not assign subcluster 0 to a specific cell type (Supplementary Fig. 9b). We next assessed the expression of secreted factors produced by SMC and fibroblast cluster and have receptors on endothelial cells across different subclusters. Most angiogenic factors including Vegfa, Vegfc, Sema3c, and Cxcl12 have a trend of increase in the fibroblast subcluster. The unknown subcluster highly expresses Ptn (Supplementary Fig. 9b). Assessing expression of Cd34 shows that fibroblast subcluster is the only subcluster which expresses Cd34 (Supplementary Fig. 9c).

## Discussion

In recent years, several studies have been performed to elucidate the origin of endothelial cells contributing to neovascularization [10]. While numerous studies have indicated that endothelial progenitor cells circulate from bone marrow and can contribute to neovasculogenesis [2, 3, 25, 27, 29, 54], recent publications suggest endogenous neovascularization is driven by tissue-resident endothelial cells which do not arise from bone marrow cells [13, 22, 32]. Our results using single-cell RNA sequencing allow us to challenge this dogma. Our approach enabled the production of single-cell resolution maps of whole BM-derived cells which migrate to the heart in hemostatic conditions and after induction of angiogenesis and arteriogenesis by episodic myocardial ischemia. Moreover, our results may explain the observation by Schaper et al. in which very few proliferating vascular cells were found in a canine model of coronary collateral growth [49]. Specifically, in coronary collaterals during their active growth phase, only 4–6% of the cells showed evidence for mitosis, which renders difficult the explanation of how a coronary collateral in a dog can increase its caliber tenfold or more through cell division alone. Perhaps the explanation for this is that collateral growth also is the result of BM cell recruitment, engraftment, and differentiation into a vascular phenotype.

Our previous results have suggested a role for bone marrow-derived cells in ischemia-induced angiogenesis and arteriogenesis [5, 9]. In the present study, we documented the differentiation of bone marrow cells into components of coronary blood and lymphatic vessels during ischemia-induced vascular growth. Specifically, we found a distinct BM-derived population with high expression of endothelial markers including Cdh5, Cav1 and Kdr from BM cells in the heart. The presence of these cells was confirmed by staining the heart sections for endothelial-specific markers. A previous study revealed contribution of Cdh5-positive cells clonal expansion into ischemia-induced neovascularization in the heart, but this study did not address the origin of the Cdh5-positive cells [36]. Our data showed that 23.5% of endothelial population express Cd34. Another group which used Cd34-CreERT2 transgenic mice provided evidence showing a low number of Cd34-positive endothelial cells in the mouse femoral artery in hemostasis which increased after vascular injury[22]. To address whether these CD34^+^ cells originate from BM, they transferred Cd34-CreERT2 BM cells to the irradiated wild-type mice and reported a scarce number of CD34 positive cells co-expressing CD31 in the femoral artery after injury [22]. Their differential gene expression analysis of all femoral artery endothelial cells between Cd34^+^ and Cd34^–^ populations showed CD34^+^ ECs had a stronger capacity of endothelium development and blood vessel morphogenesis [22]. Consistently, our findings showed BM-derived Cd34^+^ ECs were enriched with the genes involved in blood vasculature development and regulation of angiogenesis pathways.

An important facet to our work bears on the question of what signals trigger the bone marrow cells to home to the heart and differentiate into endothelial cells. Although we cannot answer that question, there are observations in the literature that provide some insights. Ischemia, the occurrence of inadequate blood flow and oxygenation in a tissue, was traditionally viewed as a local event but recent evidence reveals this view is overly simplistic. In a review by Kleinbongard et al., the authors argued that ischemia should be thought of as a systemic event, where an ischemic tissue communicates with remote organs through the interaction of neural responses, cytokines, and hormones [28]. Other evidence for remote communication in ischemia involved interactions between the spleen and vagal nerves [33, 34]. These articles focused on remote communication in ischemic preconditioning, which we believe relates to our study, as collateral growth would be an optimal event in ischemic preconditioning because it would prevent the occurrence of ischemia. Whether the recruitment of bone marrow cells to the heart involves neural responses, splenic processing, and/or cytokines remains to be determined and is an important facet to our work.

Based on the expression of putative endothelial subpopulation markers we propose a heterogenous BM-derived endothelial population in the heart. Previous studies have not revealed if BM-derived cells transdifferentiate into different populations of ECs in the heart. Single-cell transcriptome analysis of murine ECs showed high percentage of capillary ECs (59.6%), lower percentage of artery ECs (8.9%) and venous ECs (19.5%), and a lower percentage of lymphatic ECs (3.7%) [26]. We observed a similar pattern in BM-derived cardiac ECs, high percentage of capillary ECs (64%) with lower percentages of artery (11.5%), venous (4.3%), and lymphatic ECs (7.1%). We emphasize that our results were from cells homing from bone marrow, which may not reflect the overall populations.

We were surprised by the numbers of BM-derived cells homing to the heart under basal conditions, i.e., a comparison of Control and RI results. However, a comparison of 20 top-ranked genes in endothelial population of BMH-Control and BMH-RI shows differences between two groups. The BMH-RI expressed more angiogenic genes than BMH-Control ECs. This is consistent with the evidence indicating hypoxia stimulates angiogenesis [46, 62] and our previous results revealing the importance of VEGF expression in adaptations of the coronary circulation to ischemia [38, 56]. Vessel development module was enriched in capillary, capillary artery and artery subsets consistent with high expression of angiogenesis markers in these subclusters. Upon RI, BM-derived ECs activated the vessel development module.

The literature has suggested that glycolysis is the major ATP source in ECs [62]. However, a recent single-cell study, which addressed heterogeneity of ECs from different tissues showed inter-tissue heterogeneity of metabolic markers as well as heterogeneity in different EC subtypes within a single tissue. They reported that cardiac and muscle ECs had higher expression of genes associated with lipid metabolism, compared to ECs from other tissues [26]. Our metabolic gene expression analysis revealed that there was not an obvious difference in carbohydrate metabolism gene expression across BM-derived EC subpopulations, but capillary, capillary artery, and artery subsets showed high expression of genes associated with lipid metabolism. We also observed enrichment of TCA cycle in the BMH-Control ECs and lipid metabolism pathway in the BMH-RI ECs.

It was demonstrated that cardiac ECs undergo a transient mesenchymal activation after MI and return to hemostasis 10 days after a myocardial infarction [55]. We had enrichment of genes associated with a mesenchymal phenotype in vein and artery or vein ECs subpopulation, but we did not see an obvious difference in mesenchymal gene expression between BMH-Control and BMH-RI in DEG analysis. This could be related to transition nature of this process which is not detectable in long-term studies [55]. We also had activation of extracellular matrix organization module in the vein EC subsets. Mesenchymal activation is believed to be associated with metabolic adaptation with a switch from fatty acid toward a glycolytic metabolism [55]. Consistent with this observation, we had lower expression of lipid metabolism genes in the in the vein ECs subpopulation.

We observed higher Vegfa signaling in BMH-RI compared to BMH-Control ECs. A previous study showed inhibition of prox1a, Vegfc and Vegfr-3 but increased expression of Vegfa in a zebrafish model of hypoxia [12]. Interestingly, BMH-RI ECs mostly received Vegfa from Cd14^+^ macrophages. Notably, macrophages have been implicated in coronary angiogenesis and arteriogenesis [19]. Vegfc was the major component of Vegf signaling in the BMH-Control ECs. Several studies have demonstrated that Vegfa is the main regulator of angiogenesis. This growth factor can regulate angiogenesis by binding VEGFR-1 (Flt-1) and VEGFR-2 (KDR) [6, 30, 53]. On the other hand, VEGF-C/VEGF-D and their receptor, VEGFR-3 (Flt-4), plays key roles to regulate lymphangiogenesis [6, 30, 53]. Consistently we had higher percentage of BM-derived lymphatic ECs in the heart sections of Control compared to RI rats. It raises the question whether BM cells mainly tend to differentiate into lymphatic ECs under control, normoxic conditions, but shift toward other EC subtypes during hypoxia.

There are limitations in our study that affect our conclusions. Our study is subject to the inherent limitation of a small sample size, which may affect statistical power and the confidence in detecting true biological variation. We believe our findings provide valuable preliminary insights into the presence of bone marrow-derived cells in the heart under repetitive ischemic conditions. However, we acknowledge the need for caution in interpreting the results and emphasize the importance of further validation and replication in future studies. It is also important to acknowledge the limitation of our study in providing a mechanistic analysis of how bone marrow-derived cells increase blood flow in the damaged heart and how repetitive ischemia enhances these effects. Future research focusing on elucidating the underlying mechanisms behind these phenomena could provide valuable insights into therapeutic strategies for cardiovascular diseases. Investigating these mechanisms in detail will be crucial for a more comprehensive understanding of the therapeutic potential and optimization of BM-derived cell therapy for heart repair. Moreover, our study focused on one point in time in that we examined the differentiation of BMC into populations of endothelial cells at the end of our protocol (day 17 of RI). Perhaps, at earlier and even later time points, these values could be different. The use of the rat for our studies can be viewed as a limitation as well as an advantage. We have a long-standing history of studying coronary collateral growth in rats, which enables us to compare our present results to our historical studies [9, 11, 17, 18, 35, 42, 43, 45, 56, 63]. However, another approach to our study would have been to use a mouse model of coronary collateral growth, combined with lineage tracing techniques to examine of cell differentiation. Although this is conceivable, the mouse model of coronary collateral growth seems unique in that the coronary collaterals are newly formed vessels as opposed to the preexisting collateral vessels in rats, as well as other species including humans [51, 52, 59, 60]. Despite these limitations, we believe our data strongly support the concept that BMC differentiate into 7 sub-types of endothelial cells in the heart during the vascular adaptations to myocardial ischemia.

Taken together, our study revealed novel insights in contribution of BM cells to endothelial populations in the heart. Specifically, we found seven BM-derived EC populations in the heart with distinct EC subtypes and distinguished modules. Interestingly the occurrence of BM-derived endothelial cells occurred under normal conditions, implying a role for continual endothelial replenishment under basal conditions, as well as the cells contribution to the vascular adaptations in response to myocardial ischemia.

### Supplementary Information

Below is the link to the electronic supplementary material.

Supplementary file1 (DOCX 15 KB)

Supplementary file2 (PDF 4180 KB)

## Data Availability

The data are available upon request to the corresponding author.
