# Supplementary material for: Bone marrow cells contribute to seven different endothelial cell populations in the heart
Source: Basic Res Cardiol. 2024 Jul 4;119(4):699–715. doi: 10.1007/s00395-024-01065-x (PMC11319501; doi:10.1007/s00395-024-01065-x)
Supplement: Supplementary file 1 — Supplementary file1 (DOCX 15 KB) [file 395_2024_1065_MOESM1_ESM.docx]

**Supplementary Figures legends**

**Supplementary Fig. 1 Echocardiographic measurements of myocardial blood flow (MBF)** a. Representative images of contrast echocardiography assessment of MBF of rats on day 0 and 17 after initiation of repetitive ischemia. b. Representative flow cytometry plots showing the sorting of the BM cells for GFP and PI from the bone (BM) and from the heart (BMH) of BM transplanted rats. The GFP^+^ PI ^–^ BM cells were used for single cells RNA seq

**Supplementary Fig. 2 Annotation of clusters based on the expression of canonical markers.** Quality control metrics showing number of unique genes (features), number of read counts, percentage of mitochondrial gene counts in the BM cells from the bone (BM) (a) and BM cells from the heart (BMH) (b). c. Dot plot showing expression of top markers for each cluster from differential expressed genes data. Dot color represents the relative expression of genes and dot size represents -log P-value

**Supplementary Fig. 3 Abundance of BM derived proliferative cells in the heart.** Bar graph showing relative fraction of proliferative cells across different cell types in BM (a) and BMH (b) cells

**Supplementary Fig. 4 Expression of endothelial specific markers.** Heatmap of endothelial specific genes expression across different clusters

**Supplementary Fig. 5 Presence of bone marrow (BM) derived endothelial cells in the heart section.** A zoom-in original image (Fig. 3) of heart sections stained for isolectin B4 counterstained with DAPI. Scale bars represent 100 μm

**Supplementary Fig. 6 Expression of endothelial markers in endothelial cell subtypes and angiogenic markers in Control and RI groups** a. Distributed stochastic neighbor embedding (t-SNE) plot visualization of endothelial cells expressing Ptprc (Cd45). b. Gene expression heatmap of endothelial specific markers across different endothelial subclusters. Color scale: red, high expression; blue, low expression. An unpaired Student’s t test was used to compare the difference of proliferative cell proportion between Control and RI groups. c. Violin plots showing expression level of angiogenic factors in endothelial population of BM cells in the heart.

**Supplementary Fig. 7 Expression of markers in Cd34+ and Cd34- endothelial cells and Control and RI groups.** Violin plots showing expression level of tricarboxylic acid (TCA) cycle genes (a) and lipid metabolism genres (b) in BMH-Control and BMH-RI groups of endothelial population of BM derived cells in the heart. c. Heatmap showing top 20 differentially expressed genes in BM derived Cd34^+^ and Cd34^-^ endothelial cells.

**Supplementary Fig. 8 Vegf signaling in Control and RI cells.** Hierarchy plots showing autocrine and paracrine Vegf signaling between endothelial and other BM derived cells in the heart (BMH). Cell-cell communication is indicated by the edges. Edge colors are consistent with the colors of sender cells and edge weights indicate the interaction strength

**Supplementary Fig. 9 BM derived smooth muscle cell and fibroblast cluster characterization.** a. t-Distributed stochastic neighbor embedding (t-SNE) plot visualization of SMC and fibroblast subclusters. b. Heatmap showing expression of canonical markers for fibroblast, SMC and secreted factors produced by SMC and fibroblast cluster received by endothelial cells. c. Distributed stochastic neighbor embedding (t-SNE) plot visualization of Cd34 expression in SMC and fibroblast cluster
