# Supplementary material for: Bone marrow cells contribute to seven different endothelial cell populations in the heart
Source: Basic Res Cardiol. 2024 Jul 4;119(4):699–715. doi: 10.1007/s00395-024-01065-x (PMC11319501; doi:10.1007/s00395-024-01065-x)

Supplementary Fig. 1

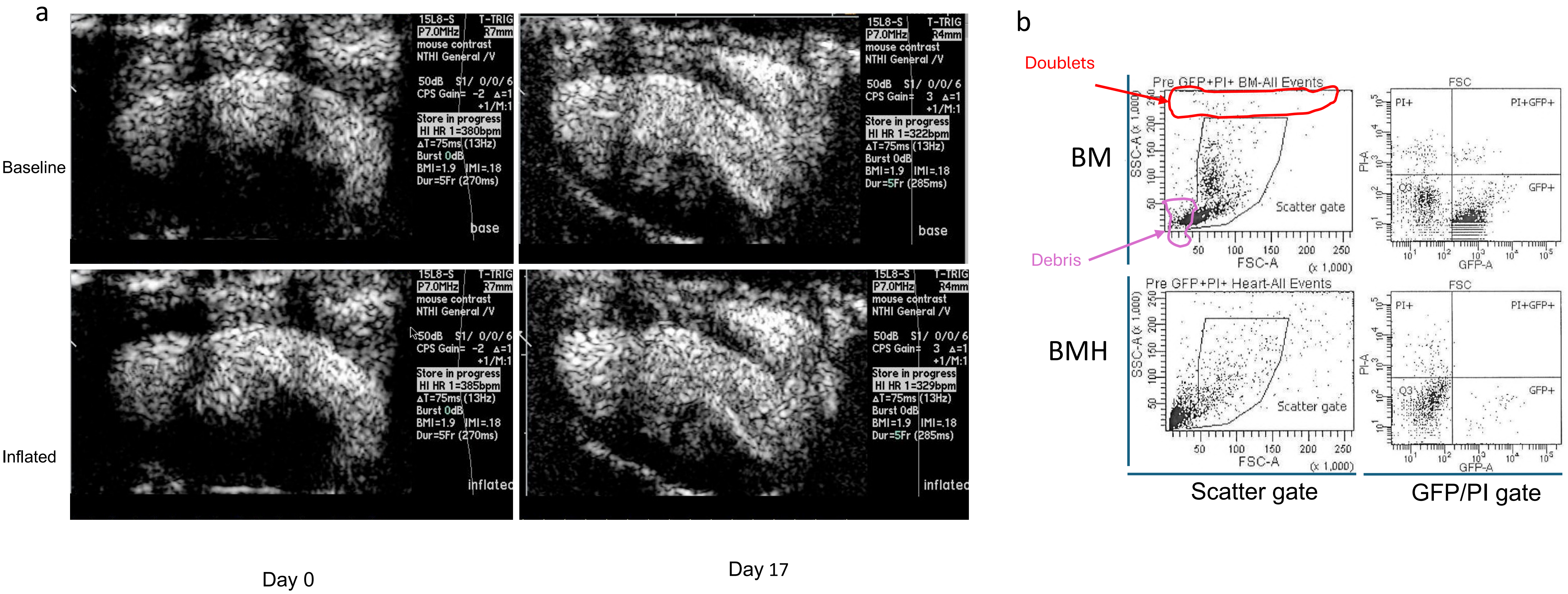

Supplementary Fig. 2

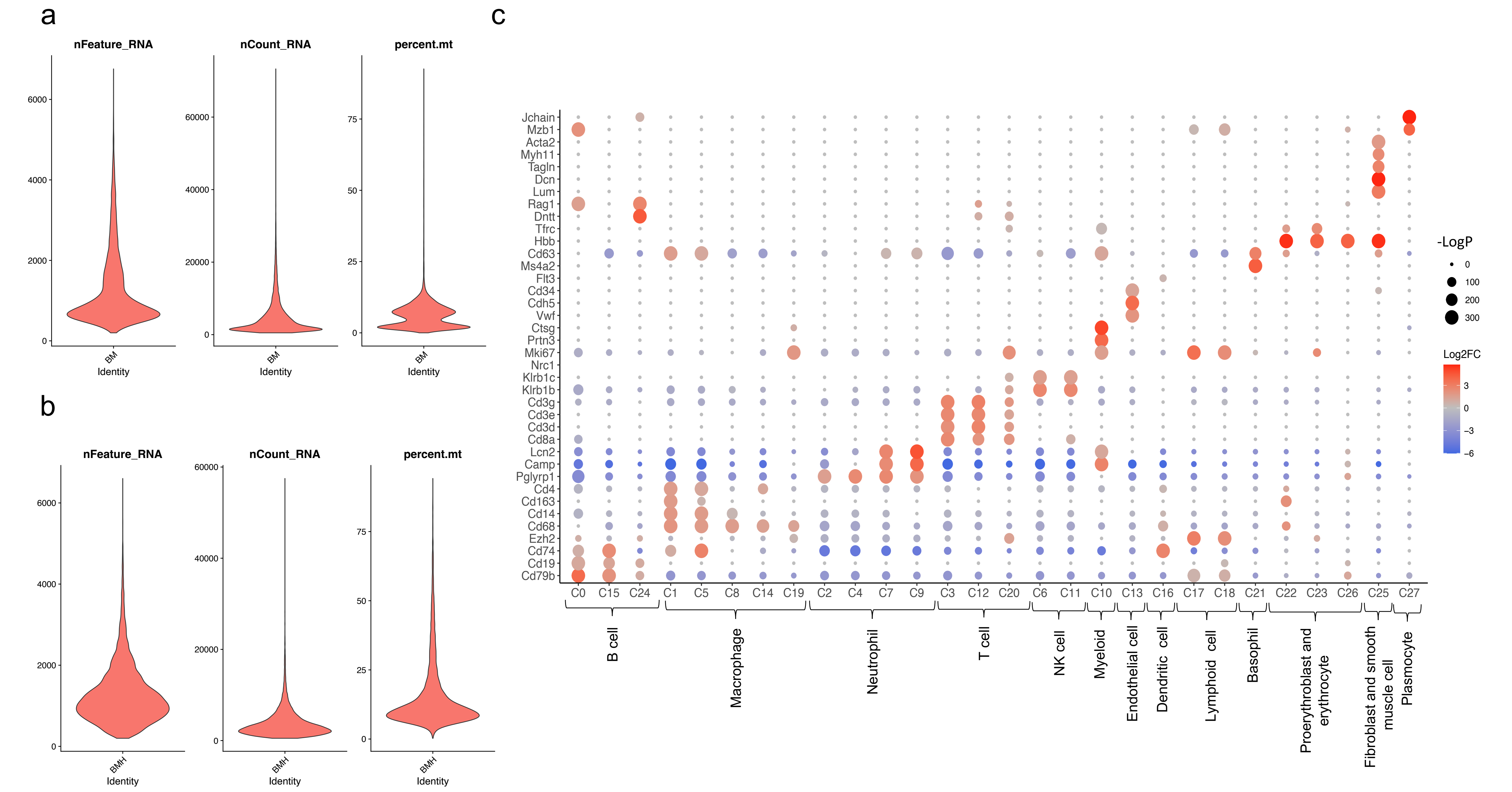

Supplementary Fig. 3

a

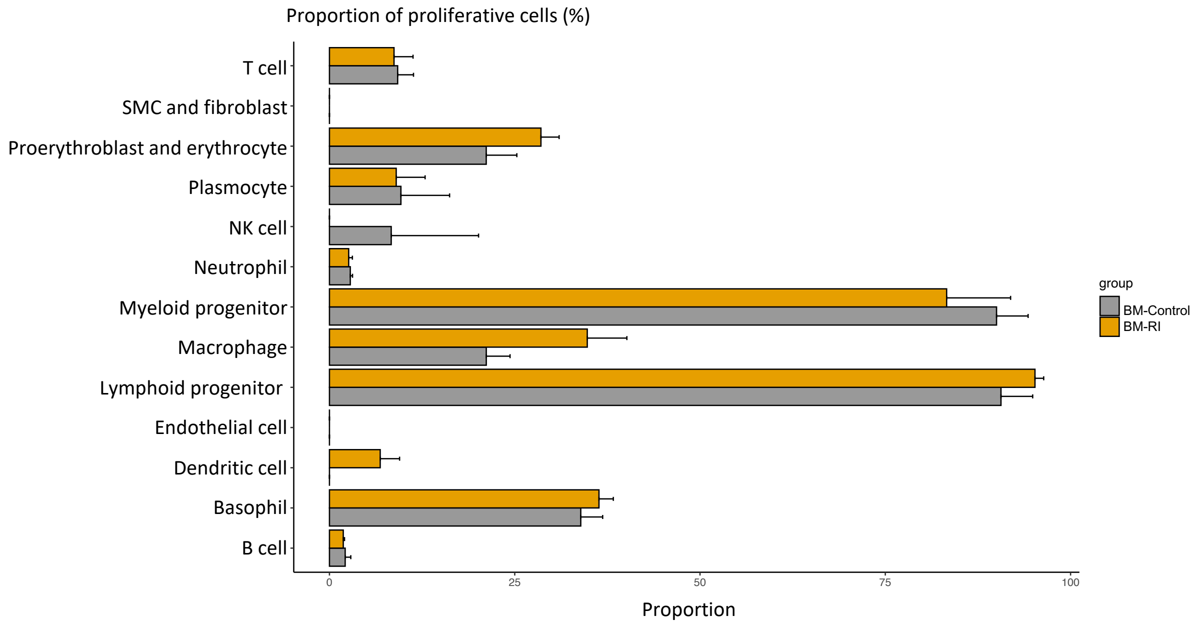

b

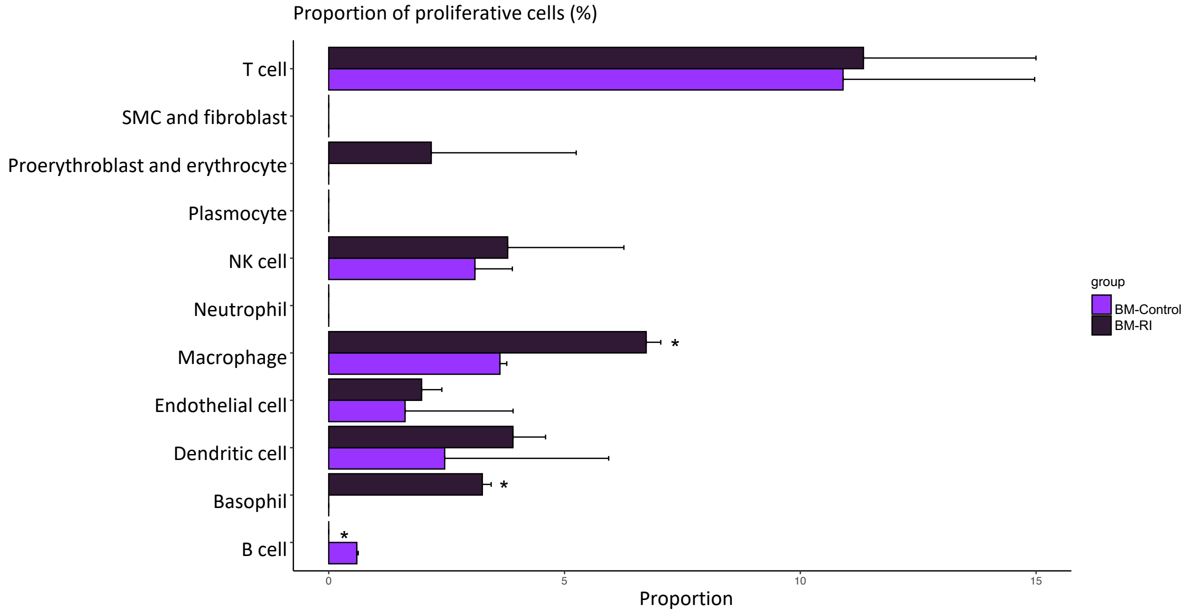

Supplementary Fig. 4

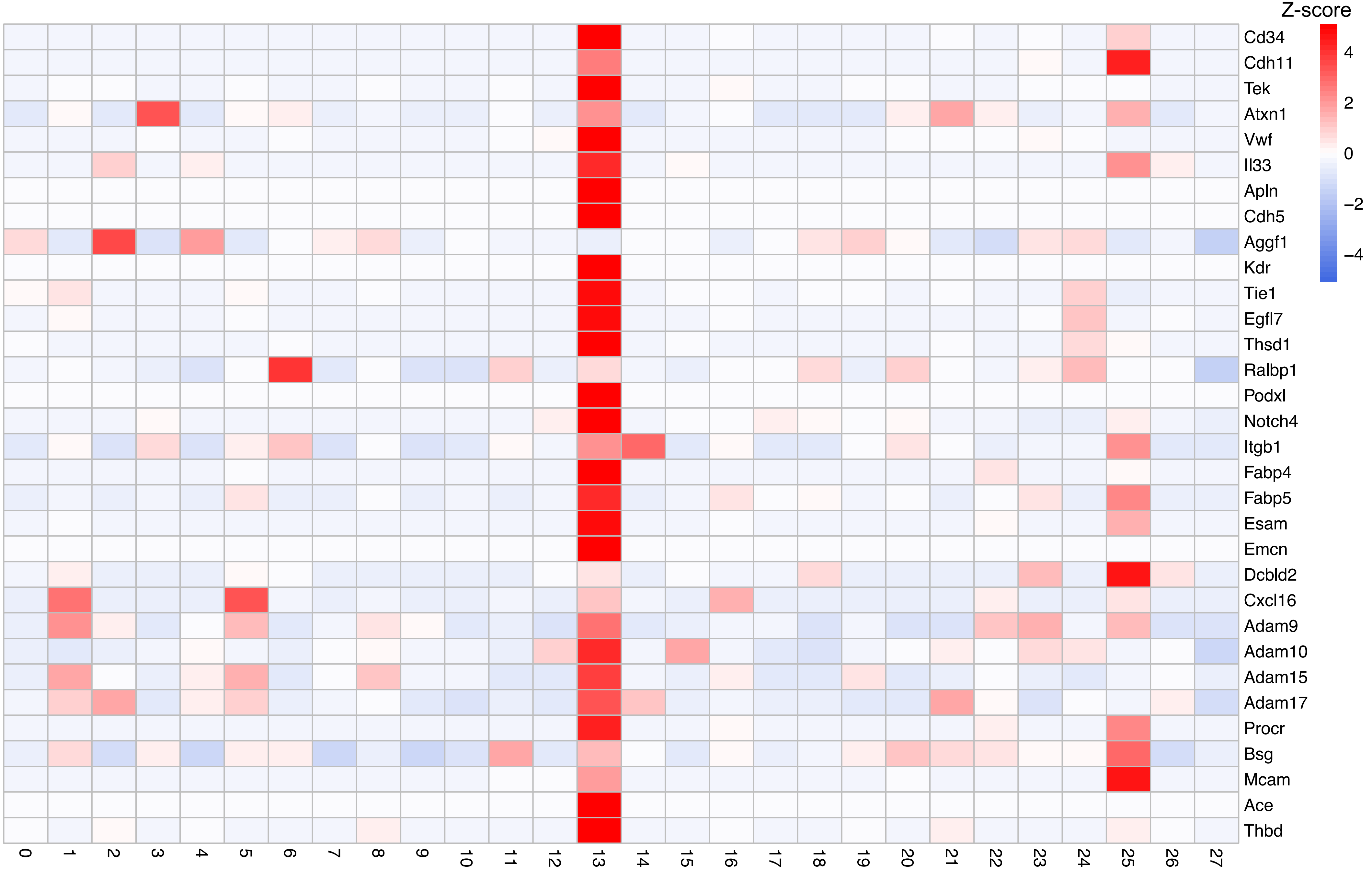

Supplementary Fig. 5

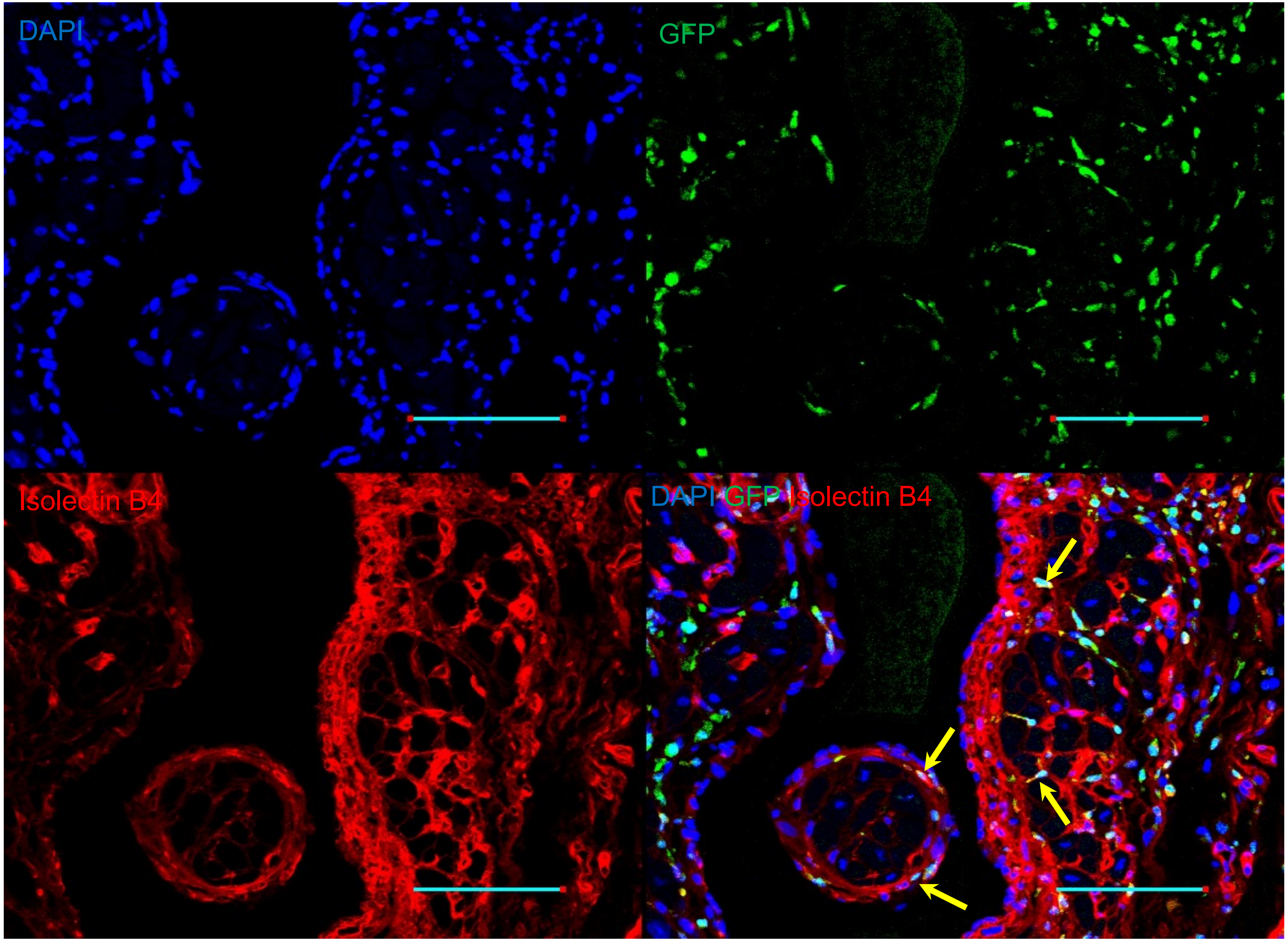

Supplementary Fig. 6

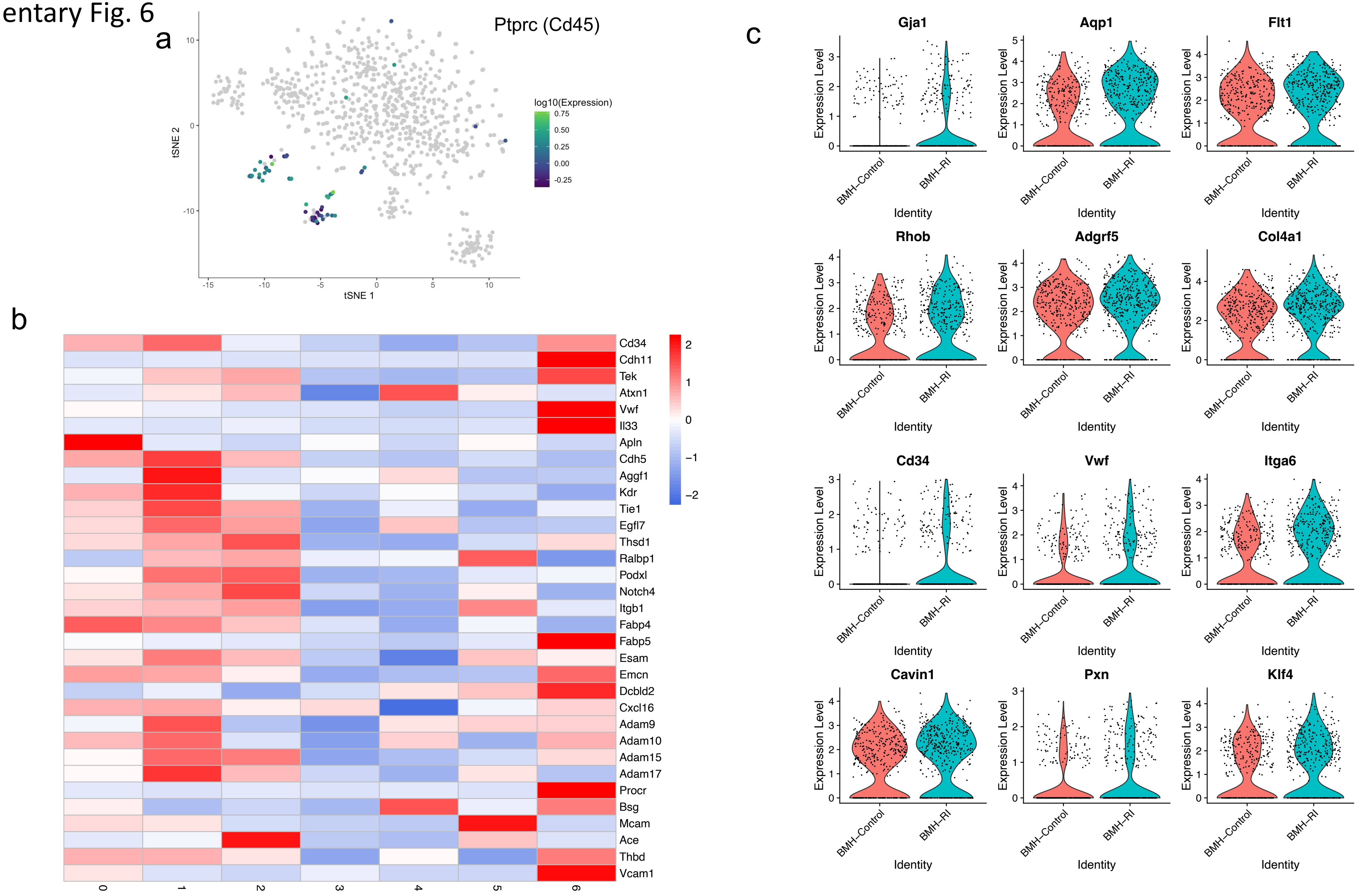

Supplementary Fig. 7

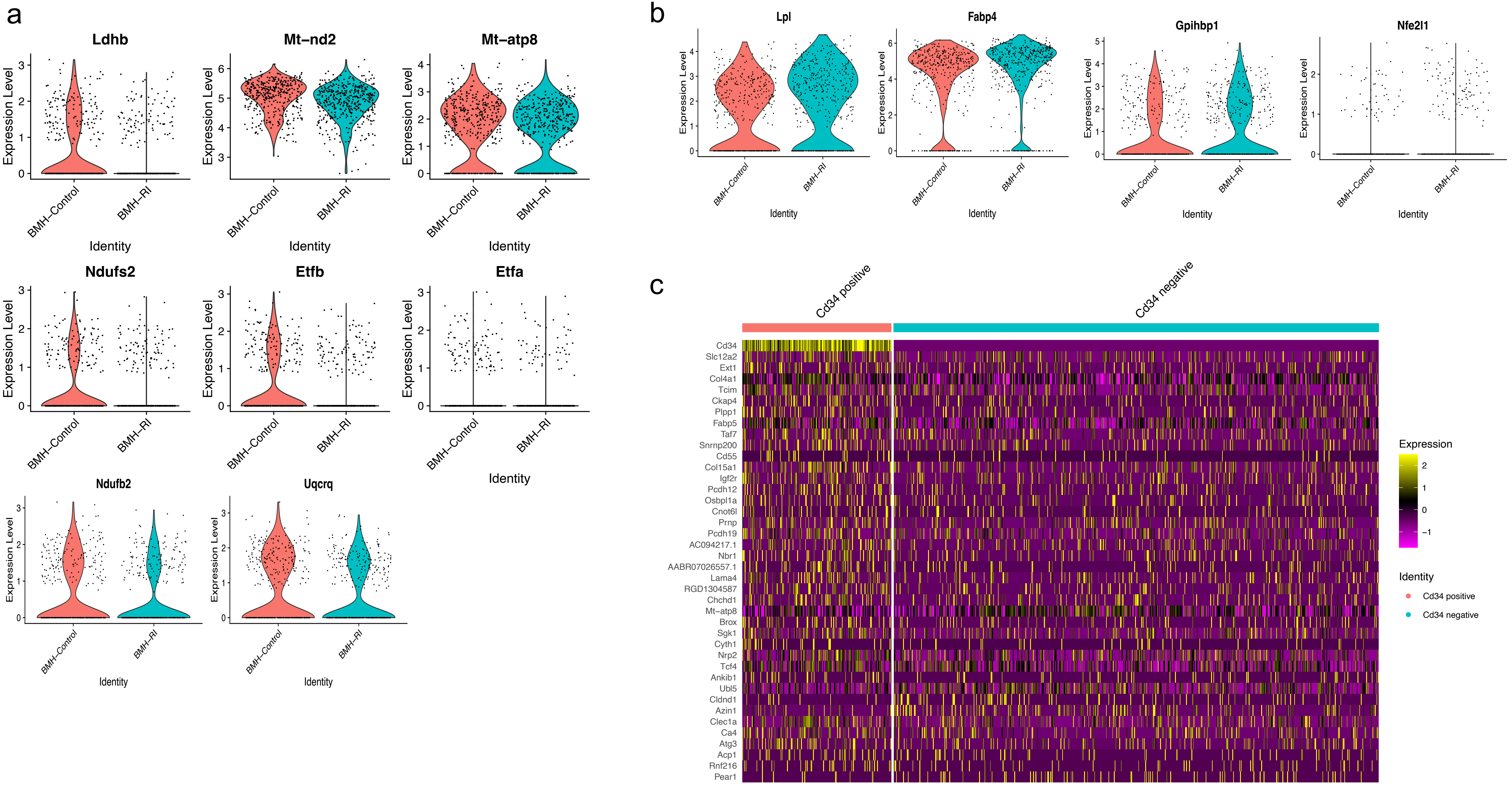

Supplementary Fig. 8

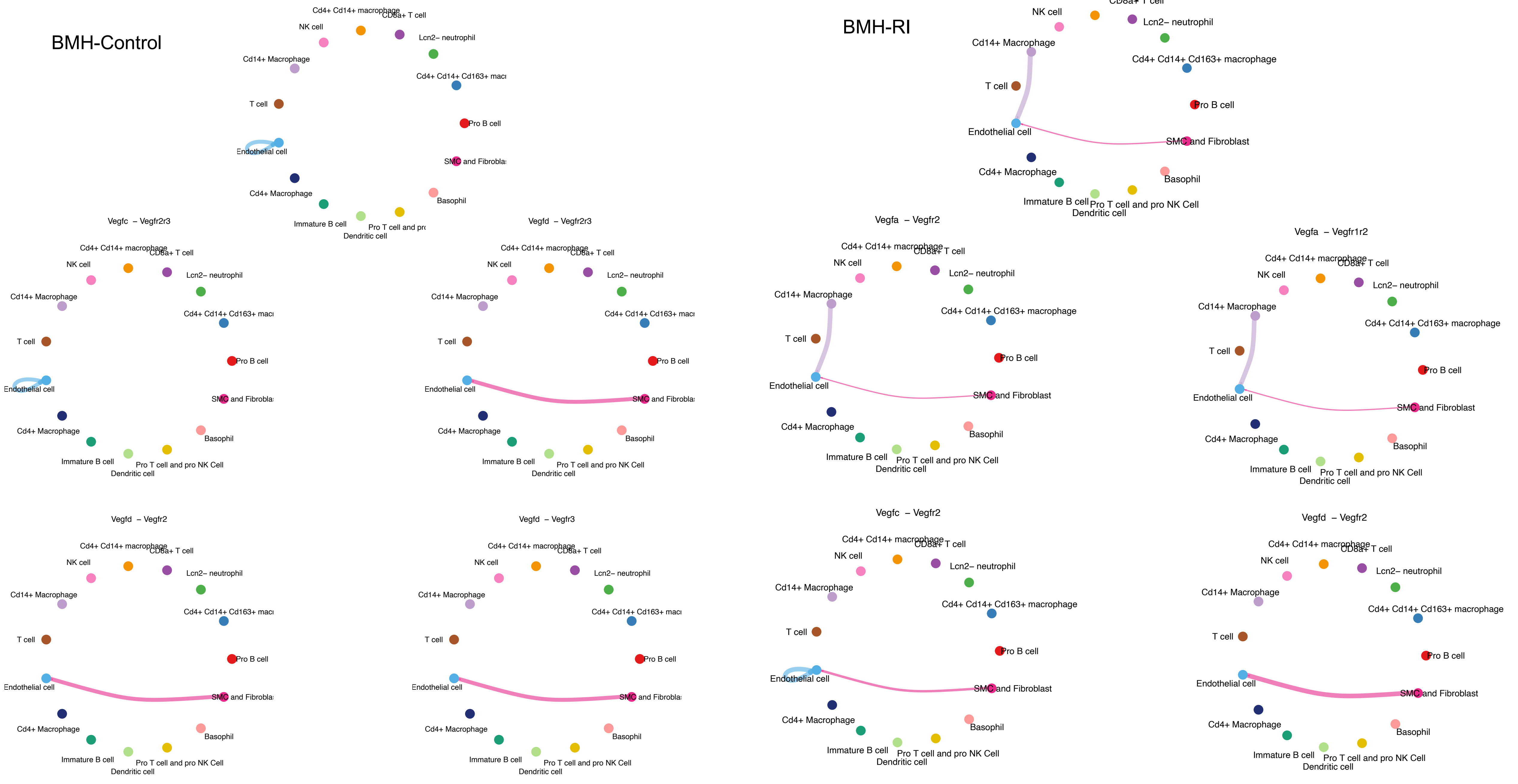

Supplementary Fig. 9

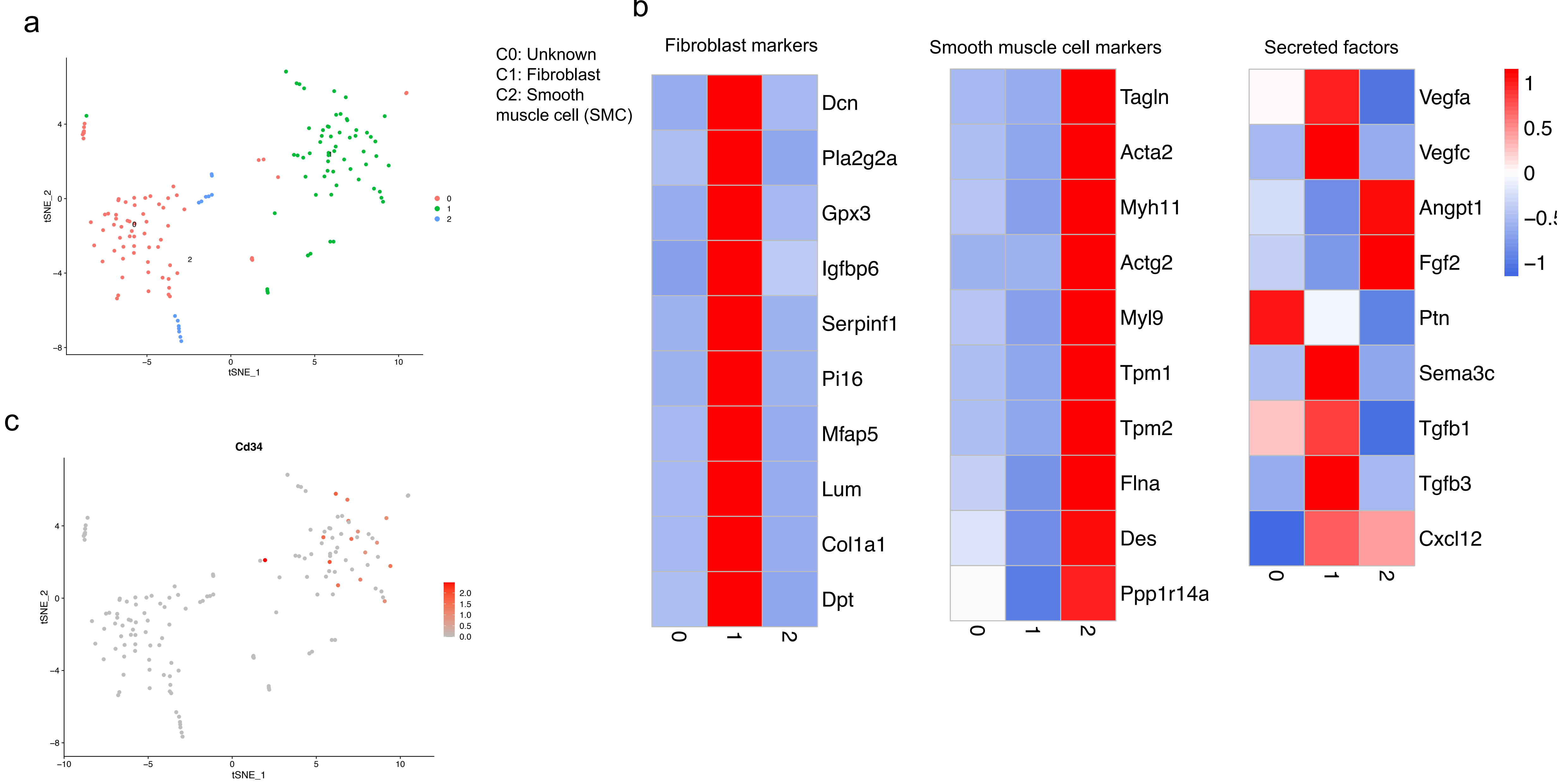

Supplement: Supplementary file 2 — Supplementary file2 (PDF 4180 KB) [file 395_2024_1065_MOESM2_ESM.pdf]
